# Targeting HER2 in breast cancer: new drugs and paradigms on the horizon

**DOI:** 10.37349/etat.2021.00037

**Published:** 2021-04-30

**Authors:** Paolo Tarantino, Stefania Morganti, Giuseppe Curigliano

**Affiliations:** 1 Division of New Drugs and Early Drug Development, European Institute of Oncology IRCCS, 20141 Milan, Italy; 2 Department of Oncology and Hematology, University of Milan, 20122 Milan, Italy; University of Southampton, UK

**Keywords:** Breast cancer, HER2, antibody-drug conjugates, tyrosine-kinase inhibitors, bispecific antibodies, immunotherapy, HER2-low

## Abstract

About 15–20% of all breast cancers (BCs) are defined human epidermal growth factor receptor 2 (HER2)-positive, based on the overexpression of HER2 protein and/or amplification of *ERBB2* gene. Such alterations lead to a more aggressive behavior of the disease, but also predict response to treatments targeting HER2. Indeed, several anti-HER2 compounds have been developed and approved in the last two decades, significantly improving our ability to cure patients in the early setting, and greatly extending their survival in the advanced setting. However, recent evolutions in this field promise to improve outcomes even further, through advancements in established HER2-targeting strategies, as well as the exploration of novel strategies. In particular, the engineering of new antibody-drug conjugates, with higher drug-to-antibody ratios (DARs) and cleavable linkers, has already led to the development of a highly effective drug, namely trastuzumab deruxtecan, recently approved by the Food and Drug Administration (FDA) and European Medicines Agency (EMA) for the treatment of advanced HER2-positive (HER2^+^) BC, and currently in study in the early setting. Moreover, the novel tyrosine kinase inhibitor tucatinib was recently approved by FDA and EMA, showing to improve survival of HER2^+^ advanced BC patients, particularly in those with brain metastasis. Immunotherapy is also being investigated in the HER2^+^ subtype, through immune-checkpoint inhibition, cancer vaccines and adoptive-cell therapies. Overall, the enlarging arsenal of promising anti-HER2 compounds is expected to deliver significant improvements in the prognosis of both early and advanced HER2^+^ BC in the years to come. Moreover, some of such agents are showing encouraging activity in the much wider population of HER2-low advanced BC patients, challenging current BC classifications. If confirmed, this new paradigm would potentially expand the population deriving benefit from HER2-targeted treatments to up to 70% of all advanced BC patients, leading to a revolution in current treatment algorithms, and possibly to a redefinition of HER2 classification.

## Introduction

The human epidermal growth factor receptor 2 (HER2) is a tyrosine kinase receptor critically involved in the carcinogenesis of the mammary gland [1]. It is a member of the HER family of tyrosine kinase receptors, including three other members [epidermal growth factor receptor (EGFR), HER3, HER4], widely interconnected into a signaling network with important implications in breast oncogenesis and clinical behavior of breast tumors [2–6]. The study of HER2 oncogenic role and the development of drugs targeting the HER2 receptor have revolutionized breast oncology in the last decades. Indeed, about 15–20% of breast cancers (BCs) harbor amplifications of the *ERBB2* oncogene, which encodes for HER2, and this alteration leads to a more aggressive behavior of disease compared to HER2-negative (HER2^–^) cases [7, 8]. However, HER2 blockade with trastuzumab and several other compounds has significantly improved the prognosis of these patients. Indeed, in the early setting, a substantial percentage of patients are cured by the addition of (neo)adjuvant HER2-blockade to standard treatment [9–11], whereas in the advanced setting the overall survival (OS) of patients has been significantly extended with the introduction of anti-HER2 agents, possibly leading to a cure for a minority of them [12, 13]. In an attempt to further improve outcomes in this subgroup of patients, multiple pharmacological strategies are being investigated, with some recent successes and with a multiplicity of promising compounds that may redesign treatment algorithms in the years to come.

The purpose of this review article is to recapitulate the landscape of novel anti-HER2 compounds under investigation, as well as explore the evolution in treatment paradigms determined by the emergence of such agents.

## Current standard of care in HER2-positive (HER2^+^) early BC

Trastuzumab is the cornerstone of treatment for patients with HER2^+^ early BC (eBC). According to seminal studies about adjuvant treatment of HER2^+^ eBC, the addition of trastuzumab to standard adjuvant chemotherapy halves the risk of recurrence, with a 10% absolute improvement in long-term disease-free survival (DFS) and a 9% increase in 10-year OS [9, 14, 15]. In these trials, trastuzumab was administered concurrently with a taxane-based chemotherapy and continued thereafter to complete 1 year of treatment. Several trials tested longer (i.e. HERA trial [16]) or shorter durations (i.e. SOLD [17], Short-HER [18], E2198 [19], FinHER [20], PHARE [21], Hellenic trial [22], Persephone [23]). Among them, only the Persephone trial was able to show the non-inferiority of the 6-month regimen. However, a recent meta-analysis including 6 large trials testing trastuzumab de-escalation, a shorter duration of adjuvant trastuzumab was statistically noninferior to its 1-year administration and resulted in lower rates of cardiotoxicity [24]. Overall, a shorter duration of trastuzumab could be discussed for patients with low-risk HER2^+^ eBC and/or for patients at high risk of toxicity, while all the others should receive the standard 12-month regimen.

As for other BC subtypes, a neoadjuvant strategy is usually preferred to the adjuvant one [25], except for very small tumors (T < 2 cm, clinically node-negative). The phase III NOAH trial first showed a strong association between pathologic complete response (pCR) in the breast and axilla and long-term outcomes [26], subsequently confirmed by further neoadjuvant trials [27]. Dual HER2-targeting with pertuzumab added to chemotherapy plus trastuzumab further increased the pCR rate [11, 28], and led to pertuzumab approval by both EMA and FDA. Of note, both studies testing trastuzumab plus pertuzumab in the neoadjuvant strategy (i.e. Neosphere [11] and TRYPHAENA [28]) were phase II trials, lacking longer-term efficacy data and unpowered for long-term outcomes like progression-free survival (PFS) and OS. In the adjuvant setting, administration of pertuzumab with trastuzumab showed only a marginal benefit in terms of invasive DFS improvement (0.9%), slightly higher in the high-risk population with node-positive and/or ER-negative HER2^+^ eBC [10]. International guidelines suggest administration trastuzumab-pertuzumab combination along with chemotherapy in high-risk patients, particularly in node-positive patients [29, 30].

Further trials recently focused on additional escalation strategies for high-risk HER^+^ eBC. The KATHERINE study demonstrated a significant benefit from the administration of trastuzumab emtansine (T-DM1) over trastuzumab in patients with residual disease after neoadjuvant therapy, and 1-year of T-DM1 is now the standard of care for these patients [31]. Extended adjuvant anti-HER2 treatment with administration of neratinib after 1 year of trastuzumab also showed a significant recurrence risk reduction, and is currently recommended for patients with hormone receptor (HR)-positive high-risk HER2^+^ eBC [32]. Oppositely, for patients considered at low risk of recurrence, a de-escalation of therapy demonstrated to be non-inferior to standard regimens. An anthracyclines-free chemotherapy with paclitaxel in combination with trastuzumab should then be considered for patients with small (T < 2–3 cm, node-negative), low-risk HER2^+^ eBC [33].

## Current standard of care in HER2^+^ advanced BC

Before the advent of anti-HER2 agents, the OS for metastatic HER2^+^ BC patients was lower than two years [34], with their treatment mostly relying on subsequent lines of traditional chemotherapy. However, the demonstration of the benefit of combining chemotherapy with the anti-HER2 antibody trastuzumab opened a new era in the treatment of this subgroup of patients [34].

Nowadays, most metastatic BC (mBC) patients receive frontline dual blockade with trastuzumab and pertuzumab combined with a taxane, followed by dual blockade maintenance (+/– endocrine treatment in tumors expressing HR) [35]. This regimen has led to an unprecedented OS of 57 months, with more than a third of the patients being alive after 8 years [36]. In case of progression to dual blockade, a standard second-line option is the antibody-drug conjugate (ADC) T-DM1, based on the results of the phase 3 EMILIA study which has proven its superiority to capecitabine plus lapatinib [37]. Nonetheless, this trial did not enroll patients pretreated with pertuzumab, thus activity of TDM-1 in this setting is still to be determined.

In contrast to the first and second line of treatment, no standard treatment is currently recommended for patients progressing to T-DM1 [35]. For about a decade, treatment in this setting has relied on the combination of capecitabine plus lapatinib [38], chemotherapy plus trastuzumab, or the two biological agents (lapatinib + trastuzumab) combined [39]. However, the recent emergence of novel anti-HER2 compounds has redesigned the field, providing highly active alternatives for T-DM1-pretreated patients [40]. More in detail, four of such agents have been recently approved by the FDA in this setting: the ADC trastuzumab deruxtecan, the Fc-engineered anti-HER2 antibody margetuximab and the tyrosine kinase inhibitors (TKIs) tucatinib and neratinib. Trastuzumab deruxtecan and tucatinib are included as treatment options in the last European School of Oncology (ESO)-European Society of Medical Oncologists (ESMO) ABC5 Guidelines [35], based on a favorable magnitude of benefit assessed with the ESMO Magnitude of Clinical Benefit Scale (MCBS) [41]. The small PFS benefit provided by neratinib in the NALA trial [42] was instead not considered enough clinically meaningful to recommend it as a treatment option, nor was the activity of margetuximab, which showed a small PFS benefit in the phase 3 SOPHIA trial [43].

## The landscape of new anti-HER2 agents

**Figure 1.**
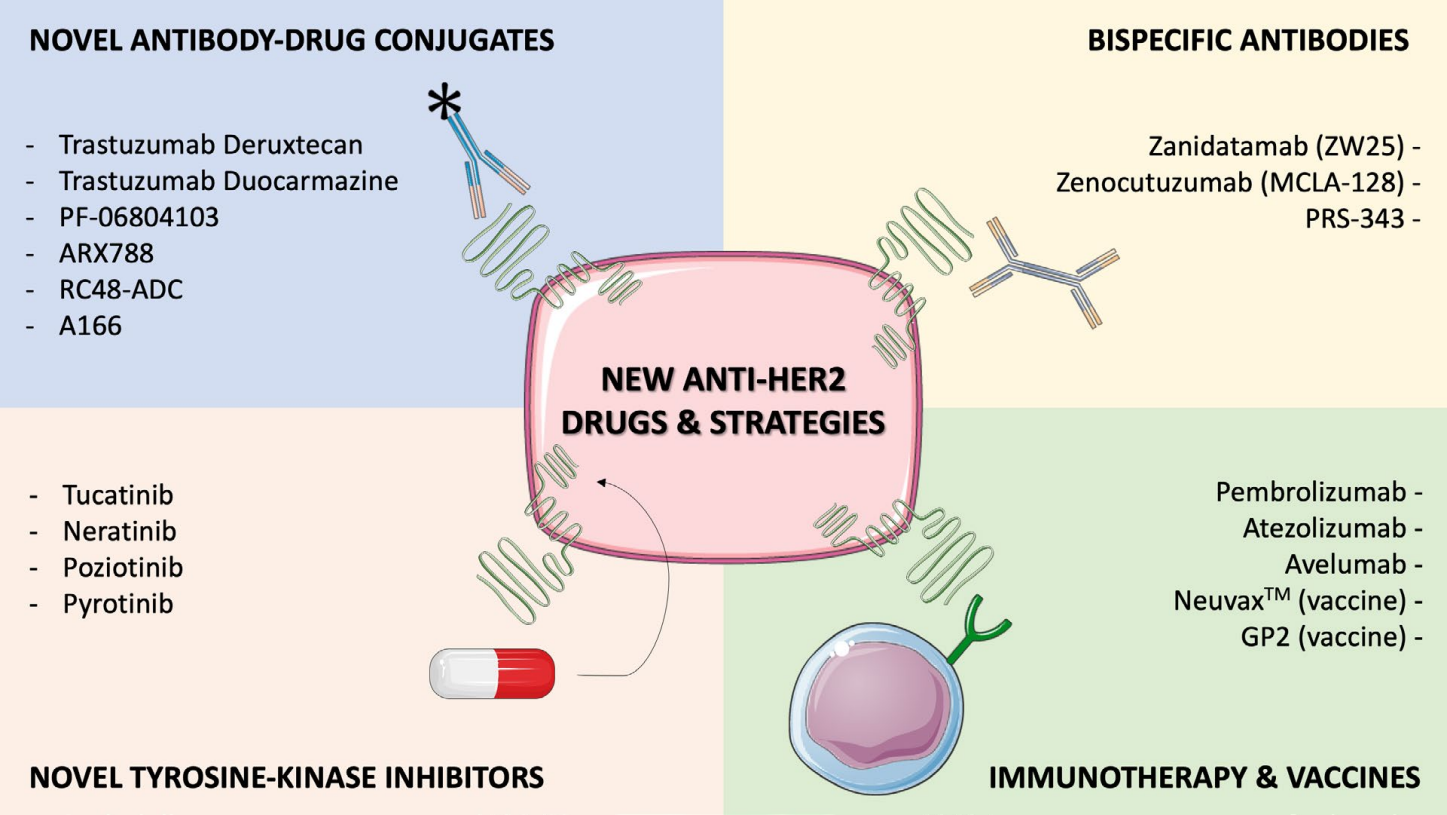
Selected, novel anti-HER2 agents or strategies under development, with available clinical data. Several drugs are currently emerging to improve outcomes in HER2^+^ BC patients, including novel ADCs, novel TKIs, bispecific antibodies (bsAbs) and immune-oncology agents. Early clinical data is available for all the compounds listed in this Figure, and for some of them confirmatory randomized trials are ongoing (e.g., trastuzumab deruxtecan, trastuzumab duocarmazine) or completed (e.g., tucatinib, neratinib). This figure was created using Servier Medical Art templates, which are licensed under a Creative Commons Attribution 3.0 Unported License; https://smart.servier.com

To further improve outcomes of mBC patients, an enlarging arsenal of new drugs targeting HER2 are being developed, encompassing various pharmacological classes (Figure 1). In this section we’ll list some of the most promising new anti-HER2 compounds under development.

### New anti-HER2 ADCs

T-DM1 was the first ADC to be developed and approved for the treatment of BC, demonstrating the potential of linking cytotoxic drugs to HER2-directed antibodies. Indeed, based on the highly differential expression of HER2 on BC cells compared to normal cells, there is a strong rationale to design conjugates able to selectively deliver potent chemotherapies in the proximity of tumor cells, in the meanwhile sparing normal tissues [44]. In this context, engineering improvements have recently led to novel ADCs, with activity in trastuzumab- and T-DM1-refractory tumors.

Three elements compose an ADC: a monoclonal antibody targeting a tumor-associated antigen (the vehicle), a chemical linker and a cytotoxic molecule (the payload). While the vehicle of novel ADCs is substantially equivalent to that of T-DM1, improvements in the linker and the payload explain the differential activity of the compounds. Indeed, in contrast with the stable thioether linker harbored by T-DM1, some novel conjugates are engineered with cleavable linkers allowing the cytotoxic molecule to diffuse outside of the targeted cell and achieve a “bystander effect”, namely the killing of neighboring non-antigen-expressing tumor cells [45, 46]. Moreover, the DARs of some novel ADCs can reach up to 8 cytotoxic molecules per antibody, whereas T-DM1 had a DAR of 3.5 [46]. Lastly, different payloads with other mechanisms of actions than inhibiting microtubules are being experimented, with particular regards to topoisomerase I inhibitors and alkylating agents.

Two ADCs have demonstrated the most relevant activity to date in T-DM1-pretreated patients. The first is trastuzumab deruxtecan, an ADC combining trastuzumab to a potent exatexan derivative through a cleavable linker, with a DAR of 8 molecules per antibody. Trastuzumab deruxtecan was tested in the phase 2 DESTINY-Breast01 trial, enrolling a cohort of highly pretreated (median: 6 lines) HER2^+^ mBC patients [47, 48]. Sixty percent of the 184 patients enrolled achieved an objective response, with an impressive median PFS 19.4 months at the last report of study outcomes [48]. Toxicities were mostly manageable and related to the chemotherapy backbone; however, about 15% of the patients experimented any-grade interstitial lung disease (ILD), with 2.7% of the cases being fatal, an observation which prompted the institution of a robust ILD monitoring and management plan for all other trials testing the compound. Based on the important activity showed by the conjugate, it was granted an accelerated approval by FDA on December 2020, pending the confirmation of the same results by the ongoing randomized phase 3 DESTINY-Breast02 trial, and more recently it was also approved by EMA. Moreover, another randomized phase 3 trial (DESTINY-Breast03) is currently comparing trastuzumab deruxtecan to T-DM1 as a second-line treatment, and results of this trial may significantly reshape treatment algorithms for this subgroup of BC patients.

The second ADC showing encouraging activity in T-DM1 pretreated patients is trastuzumab duocarmazine, which couples trastuzumab to the duocarmycin prodrug seco-DUBA (DNA alkylating agent) via a cleavable linker, with a DAR of 2.8 [45]. The drug achieved an objective response rate (ORR) of 33% in a phase 1 trial enrolling 48 highly pretreated HER2^+^ mBC patients, with a toxicity profile comparable to trastuzumab deruxtecan and mostly related to the chemotherapy backbone, although with the emergence of ocular adverse events (conjunctivitis, keratitis, dry eye) and one death due to ILD [49]. The randomized phase 3 TULIP trial is ongoing, comparing the conjugate to standard of care treatment in pretreated HER2^+^ mBC patients (NCT03262935).

A multiplicity of other anti-HER2 ADCs, listed in Table 1, is being tested in this subgroup of patients, and may further expand the treatment arsenal of pretreated HER2^+^ mBC patients.

**Table 1.** Activity of selected, novel anti-HER2 compounds under development

**Compound**	**Sponsor**	**Phase**	**BC patients treated**	**ORR in study arm**	**Median PFS in study arm**	**NCT.gov**
Monoclonal antibodies						
Margetuximab (+ chemotherapy) [43]	MacroGenics	III	536	25%	5.8 months	NCT02492711
Antibody drug conjugates						
Trastuzumab deruxtecan [47]	Daiichi Sankyo	II	184	60.9%	16.4 months	NCT03248492
Trastuzumab duocarmazine [49]	Byondis B.V.	I	48	33%	7.6 months	NCT02277717
PF-06804103 [50]	Pfizer	I	10	66%	NA	NCT03284723
ARX788 [51]	Ambrx, Inc.	I	45	31%	NA	NCT03255070
RC48-ADC [52]	RemeGen	I	70	31%	5.8 months	NCT03052634
A166 [53]	Klus Pharma Inc.	I	24	26%	NA	NCT03602079
bsAbs						
Zanidatamab (ZW25) [54]	Zymeworks Inc.	I	13	46%	NA	NCT02892123
PRS-343 [55]	Pieris Pharma, Inc.	I	16	12%	NA	NCT03330561
Zenocutuzumab (MCLA-128) (+ trastuzumab & vinorelbine) [56]	Merus N.V.	II	28	19%	NA	NCT03321981
TKIs						
Tucatinib (+ trastuzumab & capecitabine) [57]	Seagen Inc.	II R	612	40%	7.8 months	NCT02614794
Neratinib (+ capecitabine) [42]	Puma Biotech. Inc.	III R	621	33%	5.6 months	NCT01808573
Poziotinib (+ capecitabine) [58]	Spectrum Pharma Inc.	II	106	25%	4 months	NCT02659514
Pyrotinib (+ capecitabine) [59]	Jiangsu HengRui Med. Co.	II	267	67%	12.5 months	NCT03080805

R: randomized; NA: not applicable; PRS: progesterone receptors

### New anti-HER2 TKIs

The TKI lapatinib was historically the second anti-HER2 agent to be approved, showing to improve outcomes in HER2^+^ mBC patients [38]. Based on this early success, a variety of other anti-HER2 TKIs were investigated, leading to a second approval, namely neratinib for the extended adjuvant treatment of early HER2^+^ BC [32]. More recently, neratinib was also approved by FDA for the advanced setting, based on a relatively small PFS benefit demonstrated in the NALA trial [42]. Further promising TKIs showing activity in HER2^+^ mBC are the irreversible pan-HER TKIs poziotinib [58] and pyrotinib [59]. However, the most interesting recent advancement in the field was the development of tucatinib, a selective anti-HER2 TKI with a 500-fold selectivity for HER2 compared with EGFR, which allows to reduce toxicities related to EGFR inhibition.

Tucatinib was tested in the randomized HER2CLIMB trial, which enrolled 612 HER2^+^ mBC patients pretreated with trastuzumab, pertuzumab and T-DM1. In this population, the addition of tucatinib to a backbone of capecitabine and trastuzumab significantly prolonged PFS (7.8 *vs.* 5.6 months, HR = 0.54, *P* < 0.001) and OS (21.9 *vs.* 17.4 months, HR = 0.66, *P* = 0.005) [57]. Notably, almost half of the patients enrolled in the trial had brain metastases (BMs), including both stable and active BMs. In this population, the compound more than doubled the intracranial ORR (47% *vs.* 20%, *P* = 0.03) and PFS (9.9 *vs.* 4.2 months, HR = 0.32, *P* < 0.0001), and led to a numeric improvement in OS (18.1 *vs.* 12 months, HR = 0.88, *P* = 0.69) [60]. Based on these compelling results, the drug was approved by FDA for HER2^+^ mBC patients who have received ≥ 1 prior anti-HER2-based regimens in the metastatic setting, whereas the recent approval by EMA requires treatment with at least two prior anti-HER2 regimens. The TKI is also being tested in combination with T-DM1 in the HER2CLIMB-02 trial (NCT03975647), and in combination with trastuzumab deruxtecan in the HER2CLIMB-04 trial (NCT04539938).

Further promising TKIs are listed in Table 1.

### bsAbs

An emerging class of molecules in the treatment of cancer is represented by bsAbs, namely antibodies designed to bind two different epitopes or antigens [61]. These compounds are able to concomitantly block two (or more) pathways, as well as to engage immune cells or deliver payloads to cancer cells [62]. The anti-CD19/CD3 bsAb blinatumomab was the first such agent to be approved, and is available for patients affected by acute B-cell lymphoblastic leukaemia [63]. No bsAb, instead, is yet available for the treatment of patients affected by solid tumors. In the context of HER2^+^ mBC, various bsAbs are under investigation (Table 1), and some early data suggest that they may represent an effective way to improve outcomes in this population, particularly in view of their good tolerability.

The anti-HER2 agent zanidatamab (ZW25) is a bsAb binding to both ECD2 and ECD4 HER2 domains (trastuzumab- and pertuzumab-binding domains, respectively). The compound was tested in a phase 1 trial enrolling various HER2^+^ and HER2-low tumor histologies progressing to the standard of care [54]. Among 13 highly pretreated (median of 6 anti-HER2 prior regimens) HER2^+^ mBC patients, 46% achieved a partial response (PR), with a disease control rate (DCR) of 54%. Of note, the drug appeared very well tolerated, with adverse events mainly consisting in diarrhea and infusion reactions (only grade 1–2), and no treatment discontinuations. Based on this optimal tolerance, several combinatory trials have been initiated and are now ongoing, including a trial of zanidatamab plus docetaxel in HER2^+^ mBC patients (NCT04276493) and combining zanidatamab to Palbociclib and fulvestrant for HER2^+^ HR-positive mBC patients (NCT04224272). Moreover, the new compound ZW49 was obtained by the linking of a cytotoxic payload to ZW25, combining the mechanism of actions of bsAbs with ADCs, and is now being tested in a phase 1 trial (NCT03821233) [64].

Encouraging early data recently emerged also for the bsAb PRS-343, able to simultaneously bind HER2 and the costimulatory immune receptor 4-1BB on T and other immune cells [55]. PRS-343 was tested in a phase 1 trial enrolling patients with various cancer histologies, including 12 BC patients. Overall, the antibody achieved a PR in 11% of the patients and a DCR of 58%, with a pronounced post-treatment expansion of CD8^+^ T cells (more pronounced in responding patients). Similar to zanidatamab, the compound was very well tolerated, with no serious adverse events, no dose limiting toxicities and treatment-related adverse events mainly consisting in G1-2 fever, chills and diarrhea.

### Vaccines

Active immunotherapy based on anti-HER2 vaccines has been widely explored. The strong interest around these agents relies on their ability of triggering all components of the immune system, involving both the cellular and the humoral systems, as well as the innate and the adaptive components. Moreover, vaccines may induce lifelong immunity mediated by memory T cells and elicit secondary immune responses against alternative epitopes and tumor-associated antigens released by tumor cell lysis [65]. Several different HER2-directed vaccines have been investigated, including protein-, peptide-, DNA-, viral vector- and cell-based vaccines. Among them, HER2-peptides and HER2-directed dendritic cells (DCs) are the ones with more supportive data available.

E75 (nelipepimut-S) is the most studied anti-HER2 peptide-based vaccine, consisting in a human leukocyte antigen (HLA)-A2/A3-restricted peptide derived from the extracellular domain of HER2 [66]. In preliminary trials conducted in the advanced setting, E75 demonstrated to be safe and capable of inducing an effective and persistent T-cell response [67, 68], even if clinical responses were either not reported or not seen [69]. Of note, a large amount of data showed lack of efficacy for peptide vaccines in the metastatic setting [70], likely due to the high disease burden and the suppressive microenvironment that is known to impair T cells activity. The (neo)adjuvant setting seems therefore the most promising, and most of ongoing trials with HER2-direct vaccines are run in the early setting.

Initial studies carried out in the adjuvant setting showed encouraging results from the combination of E75 and GM-CSF (Neuvax^TM^). Patients with node-positive or high-risk node-negative HER2^+^ eBC were enrolled in two overlapping studies and, if HLA-A2/3-positive, vaccinated with Neuvax^TM^ [71–73]. Combined data from 108 women treated in both trials have been presented. After a prespecified follow-up of 60 months, a DFS rate of 89.7% was observed in vaccinated women, *versus* 80.2% in the control group (*P* = 0.08) [74]. Interestingly, immune responses were higher in patients with HER2-low tumors if compared to HER2^+^ eBC [75]. The randomized, placebo-controlled, phase III PRESENT study was then initiated, restricted to the HER2-low population, but recent data from a pre-planned interim analysis failed to show any benefit from vaccination, and led to early trial termination [76]. Finally, based on the rationale of a potential synergy [77], a combinatory trial of adjuvant Neuvax^TM^ plus trastuzumab in HER2-low patients was launched [78]. Two hundred and seventy-five HER2-low eBC patients, restricted for particular HLA genotypes (HLA-A2, A3, A24, and/or A26^+^), were randomized after standard therapy completion to adjuvant trastuzumab +/– Neuvax^TM^. The combination was safe, with toxicity profile being comparable in both arms. Although the vaccine wasn’t able to improve outcomes in the overall population, a planned exploratory analysis of patients with triple negative, HER2-low BC found a 74% reduction in risk of relapse in the study arm, warranting further studies restricted at this subgroup [79].

Apart from E75, other HER2-directed peptide-based vaccines have been tested in the adjuvant setting, but none of them was able to show a significant benefit in terms of disease recurrence [80].

HER2-dendritic cell vaccines are instead based on autologous DCs pulsed with HER2-peptides. Many phase I studies showed a good tolerability profile and induction of strong cell-mediated anti-HER2 immune responses, both in the metastatic and early setting [81, 82]. Interestingly, these data were confirmed also on patients with HER2^+^ ductal carcinoma *in situ* (DCIS) receiving the DC-based vaccine as neoadjuvant treatment [83–85]. Several phase II studies specifically designed to investigate anti-tumor activity of HER2-DC vaccines are ongoing (NCT03384914, NCT03630809, NCT02336984).

### Adoptive cell therapies

Adoptive T-cell therapies (ACTs) consist of isolation of autologous T-cell, their genetic engineering *ex vivo* to recognize specific tumor-antigens, their expansion and final reinfusion. Three main techniques have been developed: tumor-infiltrating lymphocytes (TILs) therapy, T-cell receptor (TCR) therapy and chimeric antigen receptor (CAR) T cell therapy. ACTs demonstrated to be highly effective in hematological malignancies, and are currently under investigation in several solid tumors, including BC.

Numerous preclinical studies have tested ACTs in HER2^+^ BC models. Both TCR and CAR T cells therapy demonstrated to be effective, inducing apoptosis in BC cells [86] and delaying tumor progression in HER2^+^ animal models [87–90]. Oppositely, little evidence is available from clinical studies on humans. A case report published in 2009 firstly described results from the adoptive transfer of autologous HER2-specific T cell clones in a patient with HER2^+^ mBC. Transferred T cells rapidly disappeared from the peripheral blood and accumulated in the bone marrow, where tumor cells were efficiently killed and disappeared after completion of ACT. Unfortunately, no penetration into solid metastases was observed [91]. At our knowledge, only two phase I trials testing HER2-directed CAR T therapy have been published. Disis and colleagues [92] evaluated safety and efficacy of HER2 specific T cells after rapid HER2 vaccinations in 19 patients with advanced HER2^+^ BC. Treatment was overall well tolerated, but no responses were observed. Lum et al. [93] investigated instead activity of activated T cells armed with anti-CD3 × anti-HER2 bsAb in 23 women with both HER2^+^ and HER2^–^ mBC. In the evaluable patients at 14.5 weeks, 2 objective responses were observed (1 partial and 1 complete response), 11 patients achieved a disease stability, whereas 9 patients had a disease progression. Further development of CAR T cells in solid tumors is mainly hampered by prohibitive costs, need for hyper-specialized centers, and concerns about safety of these therapies. Despite well tolerated in the abovementioned trials, serious and fatal adverse events led by acute cytokine release have been reported with ACTs in solid tumors [94, 95].

## Integrating immunotherapy into HER2^+^ BC treatment

BC has been traditionally classified as a poorly immunogenic tumor. Nonetheless, it is a heterogenous disease, and this heterogeneity characterizes also the immunogenic potential of BC. Triple negative and HER2^+^ BC are now recognized as the most immunogenic among BC subtypes [96], with large evidence supporting immunotherapy activity [97]. Indeed, both these subtypes are characterized by a higher programmed cell death ligand-1 (PD-L1) expression [98, 99], tumor mutational burden (TMB) [100, 101] and median percentage of TILs [102, 103], all biomarkers of immune activation.

First clinical studies with immune checkpoint inhibitors (ICIs) in HER2^+^ BC have been conducted in the metastatic setting, with disappointing results.

The JAVELIN trial [104] is a phase I study that tested avelumab monotherapy in patients with mBC, unselected for PD-L1-expression and including all subtypes. Fifteen point five percent of 168 patients enrolled had HER2^+^ BC, but no response was observed among this subgroup of patients.

The phase Ib/II PANACEA study [105] looked instead at the combination of trastuzumab and pembrolizumab in patients with trastuzumab-resistant HER2^+^ mBC. An ORR of 15% was observed in the PD-L1^+^ cohort, whereas there were no responses among PD-L1^–^ patients. Responses achieved were durable, with a median duration of response of 11.1 months. Of note, median OS was also significantly higher in the PD-L1^+^ cohort than in the PD-L1^–^ (16.1 *vs.* 7.0 months; *P* < 0.01).

Subsequently, other trials were designed to understand if the addition of ICIs could potentially increase durability of approved regimens for HER2^+^ mBC. The phase II KATE2 study investigated the combination of T-DM1 plus atezolizumab or placebo in patients progressing to prior trastuzumab and taxane-based therapy [106]. One hundred thirty three and 69 patients were randomized to receive T-DM1 plus atezolizumab or placebo, respectively, with almost half of the patients pretreated with pertuzumab in both arms. Unfortunately, this trial failed to reach its primary endpoint of PFS benefit. Overall, median PFS was similar in the two treatment arms (8.2 *vs.* 6.8 months in the atezolizumab and placebo groups, respectively; *P* = 0.33), as well as response rates. Notably, median PFS was more than doubled in the subgroup of PD-L1^+^ patients (mPFS 8.5 *vs.* 4.1 months) [106]. In the first-line setting, the addition of atezolizumab to the standard regimen of taxane, trastuzumab and pertuzumab in currently under investigation by the NRG BR004 phase III study (NCT03199885).

Briefly, all trials testing ICIs in HER2^+^ mBC presented so far were formally negative, and failed to provide a benefit in this BC subtype. Nonetheless, biomarker analysis conducted in these trials provided crucial data about the role of TILs and PD-L1 for patients’ selection. In the PANACEA trial, objective responses were observed only in the PD-L1^+^ cohort. Additionally, a preliminary subgroup analysis showed that among PD-L1^+^ tumors, responses were observed almost only in patients with stromal TILs (sTILs) > 5%, whereas few patients with PD-L1^+^/sTILs < 5% responded (ORR 39% *vs.* 5%) [105]. Similarly, the KATE2 study showed a consistent PFS benefit from the addition of atezolizumab in PD-L1^+^ tumors. Patients whose tumors showed TILs ≥ 5% had also a longer PFS with respect to patients with TILs < 5%, while the opposite was observed in the placebo arm [106].

In the (neo)adjuvant setting, the potential benefit of adding ICIs to standard regimens is also under investigation, with two randomized phase III trial ongoing. The IMpassion 050 study (NCT03726879) is comparing the combination of chemotherapy, trastuzumab and pertuzumab with or without atezolizumab. The APTneo trial (NCT03595592) instead is testing the combination of atezolizumab plus trastuzumab-pertuzumab and two different chemotherapy regimens (with or without anthracyclines), *versus* the combination of paclitaxel, carboplatin, trastuzumab and pertuzumab. The primary endpoint is pCR for the IMpassion 050, and 5-years event-free survival for the APTneo trial. Both trials are enrolling patients with HER2^+^ eBC unselected for PD-L1-status.

## New paradigms: targeting low HER2 expressions

Besides improving outcomes of HER2^+^ BC patients, some of the novel anti-HER2 agents are also revolutionizing the way we interpret HER2 expressions. Indeed, novel anti-HER2 ADCs have recently demonstrated to be active in tumors showing low HER2 expression [immunohistochemical (IHC) staining 1+ or 2+ with negative *in situ* hybridization assay], which account for about half of all BCs [107]. These tumors are not expected to be dependent on HER2 signaling pathway, and indeed HER2 blockade with trastuzumab or pertuzumab has shown to be poorly active in this setting [108, 109].

The most relevant activity to date in HER2-low mBC was observed with trastuzumab deruxtecan, which achieved a response rate of 37% and a PFS of 11 months in highly pretreated (median of 7.5 prior therapies) patients [110]. Interestingly, the drug showed a similar activity in tumors with a HER2 IHC score of 1+ and 2+, whereas ORR numerically differed based on HR expression, with luminal-like tumors showing the highest response rate. Data of trastuzumab deruxtecan in combination with nivolumab were also recently reported at San Antonio [111]. Overall, 16 highly pretreated HER2-low mBC patients were treated with the combo, achieving an ORR of 38% and a mPFS of 6.3 months. Of note, toxicity of the regimen appeared comparable with that of trastuzumab deruxtecan monotherapy, with 10% of the patients experiencing ILD. The randomized phase 3 DESTINY-Breast04 and DESTINY-Breast06 trials are currently comparing trastuzumab deruxtecan to chemotherapy in pretreated HER2-low mBC patients, and may lead to the confirmation of the HER2-low paradigm.

Trastuzumab duocarmazine was also tested in a cohort of highly pretreated HER2-low mBC patients, showing an ORR of 28–40% and a median PFS of 4–5 months (depending HR expression). However, no confirmatory trial was yet initiated with the compound in HER2-low mBC.

Preclinical observations suggest that the activity observed with the abovementioned ADCs in HER2-low cancers may be dependent on the delivery of cytotoxic payload, rather than the inhibition of the HER2 pathway [46, 112]. In this sense, HER2 may be considered a “targetable” biomarker in HER2-low cancers, whereas it could be also considered an “actionable” pathway in HER2^+^ cancers. If ongoing randomized trials confirmed this paradigm, we may expect anti-HER2 ADCs to become useful treatment options for up to 70% of all mBC patients, with an important redefinition of current BC treatment algorithms [107].

## Tackling resistance to anti-HER2 treatments

Despite impressively improving outcomes of BC patients, anti-HER2 treatments are still subject to resistance generation through several mechanisms, both in the early and advanced settings. Such mechanisms have been extensively studied, although mostly preclinically and in the context of single HER2-blockade.

HER2^+^ cancer cells can develop resistance to HER2-targeting agents by impairing drug binding to the HER2 receptor, by constitutively activating parallel or downstream signaling pathways, trough metabolic reprogramming or reduced immune system activation [113]. The first mechanism, namely impairing the adequate binding of the compound, appears particularly relevant in the context of treatment with ADCs [114]. Indeed, by lowering HER2 expression, impairing the internalization of the conjugate or the intracellular release and permanence of the payload, cancer cells may develop resistance to HER2-targeting ADCs. Some clinical confirmations of the importance of HER2 expression level come from clinical trials testing T-DM1, where higher HER2 protein of mRNA expression led to higher activity of the compound [115, 116], whereas clinical confirmations are pending for the remaining mechanisms.

Further work is required to fully understand and overcome mechanisms of resistance to traditional anti-HER2 agents, as well as to investigate resistance mechanisms to the novel anti-HER2 agents which have recently entered the clinic.

## Conclusion

In about twenty years since the approval of the first anti-HER2 agents, a multiplicity of other anti-HER2 agents have been approved, significantly improving our ability to cure BC patients in the early setting, and greatly extending their survival in the advanced setting. Recent evolutions in this field promise to improve outcomes even further, through the engineering of novel, highly active anti-HER2 TKIs and ADCs, as well as the exploration of new ways of targeting HER2, such as bsAbs, vaccines and adoptive cell therapies. Importantly, novel agents harbor the potential to extend the benefit of anti-HER2 treatments to the wide cohort of HER2-low BC patients, a population for which no anti-HER2 agent is yet approved. Large ongoing randomized trials will determine if these promises are kept, and may relevantly reshape BC treatment algorithms in the years to come.

## Abbreviations

ACTs: adoptive T-cell therapies
ADC: antibody-drug conjugate
BCs: breast cancers
bsAbs: bispecific antibodies
CAR: chimeric antigen receptor
DARs: drug-to-antibody ratios
DCs: dendritic cells
DFS: disease-free survival
eBC: early breast cancer
EGFR: epidermal growth factor receptor
HER2^–^: human epidermal growth factor receptor 2-negative
HER2: human epidermal growth factor receptor 2
HER2^+^: human epidermal growth factor receptor 2-positive
HLA: human leukocyte antigen
HR: hormone receptor
ICIs: immune checkpoint inhibitors
ILD: interstitial lung disease
mBC: metastatic breast cancer
ORR: objective response rate
OS: overall survival
pCR: pathologic complete response
PD-L1^–^: programmed cell death ligand-1-negative
PD-L1: programmed cell death ligand-1
PD-L1^+^: programmed cell death ligand-1-positive
PFS: progression-free survival
PRS: progesterone receptors
T-DM1: trastuzumab emtansine
TILs: tumor-infiltrating lymphocytes
TKIs: tyrosine kinase inhibitors

## Declarations

### Copyright

© The Author(s) 2021.
